# Doppler ultrasonography is a useful tool for the diagnosis of hemodynamics in congestive graft injury due to heart failure after liver transplantation: A case report

**DOI:** 10.1016/j.ijscr.2021.106569

**Published:** 2021-11-04

**Authors:** Kengo Sasaki, Kazuaki Tokodai, Atsushi Fujio, Shigehito Miyagi, Michiaki Unno, Takashi Kamei

**Affiliations:** Department of Surgery, Tohoku University Graduate School of Medicine, 1-1, Seiryo-machi, Aoba-ku, Sendai, Miyagi 980-8574, Japan

**Keywords:** ALT, alanine aminotransferase, AST, aspartate aminotransferase, CHDF, continuous hemodiafiltration, CVP, central venous pressure, IABP, intra-aortic balloon pumping, LDLT, living donor liver transplantation, LV, left ventricular, LVEF, left ventricular ejection fraction, POD, postoperative day, US, ultrasonography, Congestive liver injury, Doppler ultrasonography, Liver transplantation, Intra-aortic balloon pumping, Cirrhotic cardiomyopathy, Case report

## Abstract

**Introduction:**

Patients with end-stage liver disease often have cardiac dysfunction, which can be worsened by hemodynamic instability in liver transplantation, causing congestive graft injury.

**Presentation of case:**

A 28-year-old male with Wilson's disease underwent liver transplantation. The patient's history included cirrhotic cardiomyopathy and a preoperative ejection fraction of 37% on echocardiography. After liver transplantation, massive transfusion and acute renal failure led to increased central venous pressure. Doppler ultrasonography (US) showed an increase in positive components of the hepatic vein triphasic wave, followed by pulsatile changes in the portal vein waveforms and an eventual to-and-fro pattern. Laboratory data showed severe elevations of hepatocellular transaminase levels. Based on Doppler US findings, we determined liver damage was due to passive congestion caused by heart failure. Immediate initiation of continuous hemodiafiltration (CHDF) and intra-aortic balloon pumping (IABP) led to the patient's recovery from severe heart failure and graft injury.

**Discussion:**

In our case, changes in the hepatic and portal vein waveforms and marked elevation of hepatocellular transaminases implied exacerbation of heart failure caused by hepatic congestion and injury. Worsening heart failure, in turn, led to progressive liver damage as the result of hepatic passive congestion. The patient's condition was successfully managed with early initiation of CHDF and IABP.

**Conclusion:**

Doppler US can help diagnose congestive graft injury due to heart failure in liver transplant patients and should be performed during post-transplant management of patients with cardiac dysfunction.

## Highlights

• Cardiac dysfunction can worsen after liver transplantation, causing congestive graft injury.
• In the post-transplant period, it is often difficult to differentiate the cause of elevated liver transaminases.
• Doppler ultrasonography helps diagnose congestive graft injury due to heart failure.

## 1 Introduction

Patients with end-stage liver disease often exhibit cardiac dysfunction, resulting in high-output heart failure and pulmonary hypertension [1]. Additionally, hemodynamic challenges after liver transplantation can exacerbate heart failure and produce poor outcomes [2], [3], [4], [5], [6], [7], [8]. Congestive heart failure can cause hepatic congestion and injury. However, in the post-transplant period, transplant rejection, damage from surgical complications, and drug-induced liver injury must be considered in addition to heart failure induced liver congestion as the cause of elevated hepatocellular transaminase levels.

Here, we describe a patient with Wilson's disease who presented with severe liver injury after liver transplantation. Based on abdominal Doppler ultrasonography (US) findings, we identified congestive heart failure as the cause of hepatic congestion and injury, and immediately initiated appropriate treatment with renal replacement therapy and mechanical circulatory support. This work was reported in line with the SCARE criteria [9].

## 2 Presentation of case

A 28-year-old male patient, diagnosed with Wilson's disease at age 10 years developed deteriorating decompensated cirrhosis due to medication non-compliance. The patient received living donor liver transplantation (LDLT).

Preoperative laboratory data showed pancytopenia (white blood cell count, 4500/μL; red blood cell count, 245 × 10^4^/μL; hemoglobin, 7.6 g/dL; platelet count, 3.3 × 10^4^/μL), decreased liver function (albumin, 2.4 g/dL; cholinesterase, 54 mg/dL), severe coagulopathy (prothrombin time international normalized ratio, 2.05; activated partial thromboplastin time, 60.0 s), hyperbilirubinemia (total bilirubin, 7.9 mg/dL; direct bilirubin, 2.5 mg/dL), and elevated brain natriuretic peptide level (687.8 pg/mL). Hepatocellular transaminase levels were slightly elevated (aspartate aminotransferase [AST], 47 U/L; alanine aminotransferase [ALT], 20 U/L). Echocardiography revealed a low left ventricular ejection fraction (LVEF) and left ventricular (LV) dilation without obvious signs of diastolic dysfunction. Echocardiographic measurements are listed in Table 1. Cardiac catheterization showed a low LVEF (40%), elevated left ventricular end diastolic pressure (18 mmHg), and high cardiac output (cardiac indices, 5.65 L/min/m^2^ by the thermodilution method and 6.54 L/min/m^2^ by Fick's method). Pulmonary artery pressure and right atrial pressure were normal. An endomyocardial biopsy revealed no copper deposits.

**Table 1:** Echocardiographic data.

	Before LTx	POD 3	POM 29
Left cardiac parameters			
IVSd (mm)	8	7	8
PWd (mm)	10	8	9
LVDd (mm)	60	63	48
LVDs (mm)	51	60	38
LVMI (g/m^2^)	106.2		78.6
LAD (mm)	44	47	31
LAVI (mL/m^2^)	25.8		23.0
LV systolic function			
Teichholz EF (%)	31	12	43
Simpson's EF (%)	37	18	41
FS (%)	15	6	21
LV diastolic function parameters			
E (m/s)	0.84	1	0.5
A (m/s)	0.53		0.38
E/A	1.6		1.3
DcT (ms)	173	102	194
E/e′ (sep) (cm/s)	9.7	11.6	5.7
e′ (sep) (cm/s)	8.7	8.6	8.8
Right cardiac parameters			
RAD (mm)	31		36
RVDd (mid) (mm)	37		35
RVFAC (%)		30	33
TAPSE (mm)	21.4	12.1	13.8
S′ (cm/s)	14		8.9
TRPG (mmHg)	18	15	17
RAP (mmHg)	3		3
RVP (mmHg)	21		20
Valvular function			
MR	Trivial		Trivial
TR	Trivial		Trivial
PR			–

Abbreviations: A, peak velocity of late transmitral flow; DcT, deceleration time of the early diastolic wave of transmitral flow; E, peak velocity of early diastolic transmitral flow; e′ (sep), early diastolic mitral annular velocity at the interventricular septal annulus; EF, ejection fraction; FS, fractional shortening; IVSd, interventricular septum thickness at end-diastole; LAD, left atrial diameter; LAVI, left atrial volume index; LTx, liver transplantation; LVDd, left ventricular internal dimension at end-diastole; LVDs, left ventricular internal dimension at end-systole; LVMI, left ventricular mass index; MR, mitral regurgitation; POD, postoperative day; POM, postoperative month; PR, pulmonary regurgitation; PWd, posterior wall thickness at end-diastole; RAD, right atrial diameter; RAP, right atrial pressure; RVDd, right ventricular dimension at end- diastole; RVFAC, right ventricular fractional area change; RVP, right ventricular pressure; S′, tricuspid annular peak systolic velocity; TAPSE, tricuspid annular plane systolic excursion; TR, tricuspid regurgitation; TRPG, transtricuspid pressure gradient.

LDLT was performed using a right lobe graft from the patient's father. Intraoperatively, severe coagulopathy and high central venous pressure (CVP) induced massive hemorrhage; ventricular tachycardia with hypotension developed and was resolved with three attempts of intraoperative electrical cardioversion. Operating time was 1003 min; blood loss was 19,736 mL. Intraoperative transfusions with red blood cells (9520 mL), fresh frozen plasma (12,480 mL), and platelets (1200 mL) were performed.

**Fig. 1:**
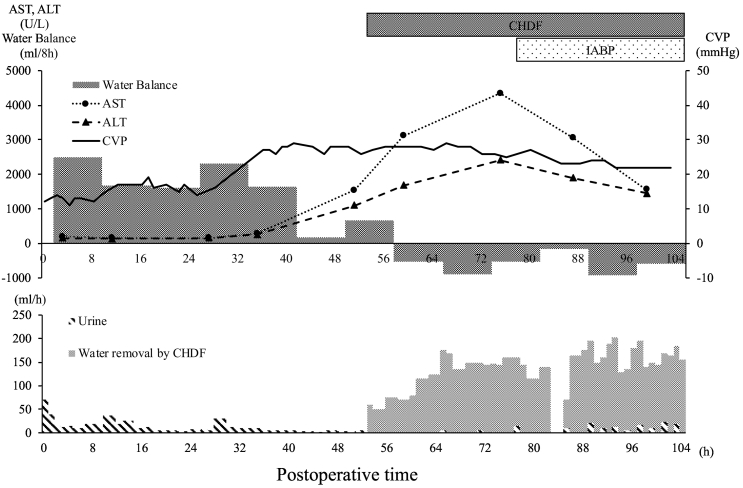
Postoperative course of water balance, CVP, and hepatocellular transaminases. Abbreviations: ALT, alanine aminotransferase; AST, aspartate aminotransferase; CHDF, continuous hemodiafiltration; CVP, central venous pressure; h, hours; IABP, intra-aortic balloon pumping.

**Fig. 2:**
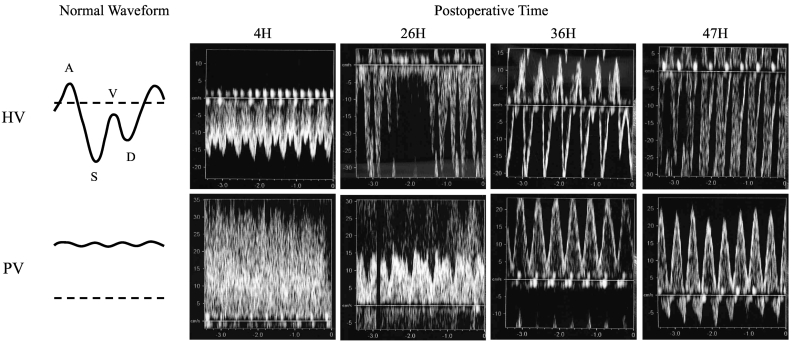
Changes observed in abdominal Doppler US; Doppler US of the right HV and the right PV in the right lobe graft. Abbreviations: H, hours; HV, hepatic vein; PV, portal vein; US, ultrasonography.

**Fig. 3:**
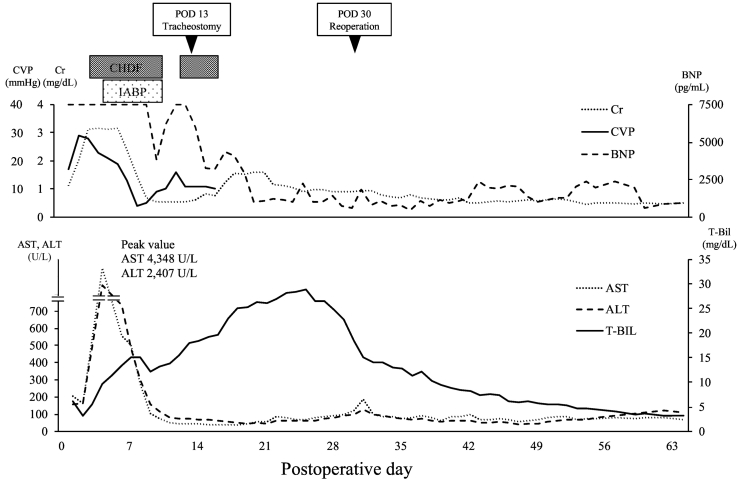
Postoperative course in the ICU. Abbreviations: ALT, alanine aminotransferase; AST, aspartate aminotransferase; BNP, brain natriuretic peptide; CHDF, continuous hemodiafiltration; Cr, creatinine; CVP, central venous pressure; IABP, intra-aortic balloon pumping; ICU, intensive care unit; T-Bil, total bilirubin.

Post-procedural abdominal hemorrhage persisted until postoperative day (POD) 2 and required massive transfusion. The patient developed acute renal failure with a marked increase in water balance and a gradual increase in CVP (Fig. 1). As CVP increased, Doppler US showed an increase in the positive components of the hepatic vein triphasic wave, followed by a change in the portal vein waveforms to a pulsatile pattern and an eventual to-and-fro pattern (Fig. 2). Laboratory data showed marked elevation of hepatocellular transaminase levels (peak values, AST [4348 U/L] and ALT [2407 U/L]). Based on these findings, we suspected exacerbation of heart failure caused by hepatic congestion and injury. Continuous hemodiafiltration (CHDF) was initiated on POD 3 to remove body water. Echocardiography showed that the LVEF had decreased to 18% on POD 3 (Table 1). Repetitive transient paroxysmal supraventricular tachycardia ensued and caused hemodynamic instability. Therefore, intra-aortic balloon pumping (IABP) was initiated on POD 4 to provide circulatory support and allow the heart to rest. Congestive heart failure and liver injury gradually improved with CHDF; IABP was withdrawn on POD 10, and CHDF was discontinued on POD 13. On POD 30, a reoperation was performed for an infected abdominal hematoma. Subsequently, the patient's general condition improved (Fig. 3). The patient was discharged from the intensive care unit on POD 63 and from the hospital on POD 95. Echocardiography at postoperative month 29 showed improvement of LVEF and LV dilation (Table 1). At 2.5 years after discharge, no cardiac event had occurred.

## 3 Discussion

Over the past few decades, advances in surgical techniques, immunosuppressants, and peritransplant management have improved patient outcomes after liver transplantation [10], [11], [12]. This has contributed to an increase in reports detailing challenging hepatic transplants with expanded indications or with multi-organ disorders. Cardiac dysfunction contributes significantly to increased morbidity and mortality after liver transplantation. Previous studies reported that conditions such as LV hypertrophy [8], diastolic/systolic dysfunction [2], [3], [4], [5], [7], right ventricular dysfunction [6], and pulmonary hypertension [13] can worsen liver transplantation outcomes. Our patient had severe systolic dysfunction and low preoperative LVEF, and was considered a high-risk case. Cardiac catheterization findings and lack of myocardial copper deposits pointed to cirrhotic cardiomyopathy as the cause of cardiac dysfunction, rather than copper deposition due to Wilson's disease. As described in other reports [14], [15], cirrhotic cardiomyopathy can improve after liver transplantation. Our patient's LVEF and LV dilation gradually improved post-transplantation.

Abdominal Doppler US is the primary method of detecting complications after liver transplantation [16], [17], [18]. The normal hepatic vein Doppler US waveform is triphasic and represents physiological changes in blood flow during the cardiac cycle. This waveform consists of four components (A, S, V, and D waves) that correspond to atrial contraction, ventricular systole, atrial overfilling, and tricuspid valve opening, respectively (Fig. 2). The normal portal vein Doppler waveform shows a continuous flow pattern. A pulsatile portal vein waveform may be abnormal; however, it is often difficult to differentiate the causes of this waveform in the post-transplant period. Decreased portal vein flow due to dehydration or portal vein stenosis, or increased hepatic sinusoidal resistance due to transplant rejection, fibrosis, or hepatic vein stenosis can cause arterial-dominant graft inflow, and a pulsatile portal vein waveform due to transmission of hepatic artery pressure [19], [20], [21], [22]. When such conditions are suspected, water volume loading is performed to improve graft blood flow and prevent thrombosis. In patients with congestive heart failure, elevated right atrial pressure is transmitted across hepatic sinusoids, resulting in a pulsatile portal vein pattern [23], [24]. In such cases, removal of excess water and circulatory support are essential treatments. It is critically important to differentiate between these etiologies, since this treatment may be contraindicated, depending on the cause of a pulsatile portal vein waveform. As our patient's water balance and CVP increased, the hepatic vein A and V waveforms increased and the portal vein waveform changed from continuous to pulsatile. These observations enabled us to identify passive congestion due to worsening heart failure as the cause of progressive liver damage. Changes of CVP and hepatic vein waveform are key findings that differentiate between passive congestion and other causes of a pulsatile portal vein waveform.

Following liver transplantation, passive congestion caused by heart failure can lead to severe liver injury; early diagnosis and immediate treatment of such patients are essential to prevent graft failure. In our patient, CHDF and IABP had an immediate and significant ameliorating effect on liver congestion. CHDF removes excess water and lessens cardiac and hepatic congestion. IABP supports circulation, stabilizes blood pressure, and allows the heart to rest. Palanisamy et al. [25] first reported the use of IABP in postoperative management of liver transplantation. Because it can cause complications such as hemorrhage, thromboembolism, and catheter infection, IABP is not the preferred treatment method. However, as delayed treatment can lead to graft failure, early initiation of these treatments should be considered.

## 4 Conclusion

Abdominal Doppler US can aid the diagnosis of congestive liver graft damage and should be performed during post-transplant management of patients with cardiac dysfunction. If signs of congestive graft injury are present, immediate appropriate treatment is necessary to prevent graft failure.

## Funding

This research did not receive any specific grant from funding agencies in the public, commercial, or not-for-profit sectors.

## Ethical approval

This work does not require a deliberation by the ethics committee.

## Consent

Written informed consent was obtained from the patient for the publication of this case report and accompanying images. A copy of the written consent is available for review by the Editor-in-Chief of this journal on request.

## Author contribution

Kengo Sasaki collected the clinical data and wrote the manuscript.

Kazuaki Tokodai, Atsushi Fujio, Shigehito Miyaagi, Michiaki Unno, and Takashi Kamei were attending doctors who performed clinical treatment, including surgical operations.

All authors have read and approved the final manuscript.

## Research registration

Not applicable.

## Guarantor

Kengo Sasaki.

## Provenance and peer review

Not commissioned, externally peer-reviewed.

## Declaration of competing interest

The authors have no conflicts of interest to declare.

## References

[1] Liu H., Jayakumar S., Traboulsi M., Lee S.S. (2017). Cirrhotic cardiomyopathy: implications for liver transplantation. Liver Transpl..

[2] Dowsley T.F., Bayne D.B., Langnas A.N., Dumitru I., Windle J.R., Porter T.R. (2012). Diastolic dysfunction in patients with end-stage liver disease is associated with development of heart failure early after liver transplantation. Transplantation.

[3] Josefsson A., Fu M., Allayhari P., Björnsson E., Castedal M., Olausson M. (2012). Impact of peri-transplant heart failure & left-ventricular diastolic dysfunction on outcomes following liver transplantation. Liver Int..

[4] Ruíz-del-Árbol L., Achécar L., Serradilla R., Rodríguez-Gandía M.Á., Rivero M., Garrido E. (2013). Diastolic dysfunction is a predictor of poor outcomes in patients with cirrhosis, portal hypertension, and a normal creatinine. Hepatology.

[5] Mittal C., Qureshi W., Singla S., Ahmad U., Huang M.A. (2014). Pre-transplant left ventricular diastolic dysfunction is associated with post transplant acute graft rejection and graft failure. Dig. Dis. Sci..

[6] Kia L., Shah S.J., Wang E., Sharma D., Selvaraj S., Medina C. (2013). Role of pretransplant echocardiographic evaluation in predicting outcomes following liver transplantation. Am. J. Transplant..

[7] Moon Y.J., Kim J.W., Bang Y.S., Lim Y.S., Ki Y., Sang B.H. (2019). Prediction of all-cause mortality after liver transplantation using left ventricular systolic and diastolic function assessment. PLoS One.

[8] Batra S., Machicao V.I., Bynon J.S., Mehta S., Tanikella R., Krowka M.J. (2014). The impact of left ventricular hypertrophy on survival in candidates for liver transplantation. Liver Transpl..

[9] Agha R.A., Franchi T., Sohrabi C., Mathew G., Kerwan A., For the SCARE Group (2020). The SCARE 2020 guideline: updating consensus Surgical CAse REport (SCARE) guidelines. Int. J. Surg..

[10] Makuuchi M. (2019). Living donor liver transplantation: looking back at my 30 years of experience. Surg. Today.

[11] Miyagi S., Kakizaki Y., Shimizu K., Miyazawa K., Nakanishi W., Hara Y. (2018). Arterial and biliary complications after living donor liver transplantation: a single-center retrospective study and literature review. Surg. Today.

[12] Tokodai K., Miyagi S., Nakanishi W., Nishimura R., Fujio A., Goto M. (2020). Characteristics and predictive value for graft fibrosis of the complement-binding capacity of donor-specific human leukocyte antigen antibodies after pediatric liver transplantation. Pediatr. Transplant..

[13] Krowka M.J., Plevak D.J., Findlay J.Y., Rosen C.B., Wiesner R.H., Krom R.A. (2000). Pulmonary hemodynamics and perioperative cardiopulmonary-related mortality in patients with portopulmonary hypertension undergoing liver transplantation. Liver Transpl..

[14] Torregrosa M., Aguadé S., Dos L., Segura R., Gónzalez A., Evangelista A. (2005). Cardiac alterations in cirrhosis: reversibility after liver transplantation. J. Hepatol..

[15] Chen Y., Chan A.C., Chan S.C., Chok S.H., Sharr W., Fung J. (2016). A detailed evaluation of cardiac function in cirrhotic patients and its alteration with or without liver transplantation. J. Cardiol..

[16] Segel M.C., Zajko A.B., Bowen A., Skolnick M.L., Bron K.M., Penkrot R.J. (1986). Doppler ultrasound as a screen for hepatic artery thrombosis after liver transplantation. Transplantation.

[17] Lomas D.J., Britton P.D., Farman P., Coulden R., Egan A., Jamieson G.N. (1992). Duplex Doppler ultrasound for the detection of vascular occlusion following liver transplantation in children. Clin. Radiol..

[18] Kok T., Slooff M.J., Thijn C.J., Peeters P.M., Verwer R., Bijleveld C.M. (1998). Routine Doppler ultrasound for the detection of clinically unsuspected vascular complications in the early postoperative phase after orthotopic liver transplantation. Transpl. Int..

[19] Wachsberg R.H., Bahramipour P., Sofocleous C.T., Barone A. (2002). Hepatofugal flow in the portal venous system: pathophysiology, imaging findings, and diagnostic pitfalls. Radiographics.

[20] Tang S.S., Shimizu T., Kishimoto R., Kodama Y., Miyasaka K. (2001). Analysis of portal venous waveform after living-related liver transplantation with pulsed Doppler ultrasound. Clin. Transpl..

[21] Sugimoto H., Kaneko T., Marui Y., Inoue S., Seo T., Hatsuno T. (2001). Reversal of portal flow after acute rejection in living-donor liver transplantation. J. Hepato-Biliary-Pancreat. Surg..

[22] Gorka W., Gorka T.S., Lewall D.B. (1998). Doppler ultrasound evaluation of advanced portal vein pulsatility in patients with normal echocardiograms. Eur. J. Ultrasound.

[23] Rengo C., Brevetti G., Sorrentino G., D'Amato T., Imparato M., Vitale D.F. (1998). Portal vein pulsatility ratio provides a measure of right heart function in chronic heart failure. Ultrasound Med. Biol..

[24] Hu J.T., Yang S.S., Lai Y.C., Shih C.Y., Chang C.W. (2003). Percentage of peak-to-peak pulsatility of portal blood flow can predict right-sided congestive heart failure. World J. Gastroenterol..

[25] Palanisamy A.P., Nadig S.N., Chedister G.R., Dowden J.E., Koch D.G., Stoll W.D. (2017). Use of intra-aortic counterpulsation in cardiogenic shock post-liver transplantation. Clin. Transpl..

